# Is postural dysfunction related to sarcopenia? A population-based study

**DOI:** 10.1371/journal.pone.0232135

**Published:** 2020-05-11

**Authors:** Angela Yun Kim, Jung Kyu Lee, Shin Hye Kim, June Choi, Jae Jun Song, Sung Won Chae

**Affiliations:** 1 Department of Otorhinolaryngology-Head and Neck Surgery, Korea University College of Medicine, Seoul, Korea; 2 Department of Otorhinolaryngology-Head and Neck Surgery, Inje University College of Medicine, Busan, Korea; Ehime University Graduate School of Medicine, JAPAN

## Abstract

Postural dysfunction is one of the most common community health symptoms and frequent chief complaints in hospitals. Sarcopenia is a syndrome characterized by degenerative loss of skeletal muscle mass, muscle quality, and muscle strength, and is the main contributor to musculoskeletal impairment in the elderly. Previous studies reported that loss of muscle mass is associated with a loss of diverse functional abilities. Meanwhile, there have been limited studies concerning postural dysfunction among older adults with sarcopenia. Although sarcopenia is primarily a disease of the elderly, its development may be associated with conditions that are not exclusively seen in older persons. Also, recent studies recognize that sarcopenia may begin to develop earlier in life. The objective of this paper was to investigate the association between the prevalence of sarcopenia and postural dysfunction in a wide age range of adults using data from a nationally representative cohort study in Korea. Korean National Health & Nutrition Exhibition Survey V (KNHANES V, 2010–2012) data from the fifth cross-sectional survey of the South Korean population performed by the Korean Ministry of Health and Welfare were used. Appendicular skeletal muscle mass (ASM)/height (ht)^2^ was used to define sarcopenia, and the Modified Romberg test using a foam pad (“foam balance test”) was performed to evaluate postural dysfunction. ASM/ht^2^ was lower in women and significantly decreased with age in men. Subjects with sarcopenia were significantly more likely to fail the foam balance test, regardless of sex and age. Regression analysis showed a significant relationship between sarcopenia and postural dysfunction (OR: 2.544, 95% CI: 1.683–3.846, *p*<0.001). Multivariate regression analysis revealed that sarcopenia (OR: 1.747, 95% CI: 1.120–2.720, *p* = 0.014) and age (OR: 1.131, 95% CI: 1.105–1.158, *p*<0.001) are independent risk factors for postural instability. In middle age subjects, the adjusted OR for sarcopenia was 3.344 (95% CI: 1.350–8.285) (*p* = 0.009). The prevalence of postural dysfunction is higher in sarcopenia patients, independent of sex and age.

## Introduction

Dizziness and vertigo (a sensation of spinning and loss of balance) are the most common symptoms experienced in a community population and the most common symptoms presented at emergency departments and primary care clinics [1–4]. The prevalence of dizziness rises with age and is about 2–3 times higher in women than in men [5]. Furthermore, dizziness is known to be associated with declining quality of life and increased functional disability [6, 7]. Most elderly individuals with long-term dizziness problems also experience balance disorders and gait abnormalities [8]. Postural dysfunction is defined as the disability of maintaining, achieving or restoring a state of balance during any posture or activity [9]. Any cognitive, sensory, or motor impairment can result in a deficit of postural dysfunction, which is a strong predictor of slip and fall accidents, a leading cause of accidental death in people older than 65 years [8–10]. Thus, the social and medical costs derived from dizziness and vertigo should be considered seriously.

Loss of muscle mass in the elderly can affect up to one-third of their former muscle mass and is known to cause gait disorders and eventually fall-related accidents [11]. Sarcopenia is a degenerative loss of skeletal muscle mass (0.5–1% loss per year after the age of 50), muscle quality, and muscle strength associated with aging [12]. The number of muscle fibers in elderly individuals is generally lower than that in younger people, especially in the lower limbs [13–15]. Sarcopenia is also considered a main contributor to musculoskeletal impairments, such as dysfunction of muscle innervation and neuronal muscle control [16, 17]. These finally influence postural maintenance, and performing functional activities requiring strength for balance and postural control becomes problematic [18].

Although sarcopenia is primarily a disease of the elderly, its development may be associated with conditions that are not exclusively seen in older persons [19]. Mainstream of studies on sarcopenia focused on elder populations over 60 or 65 years. However, recent studies recognize that sarcopenia may begin to develop earlier in life, and that variable causes beyond aging contribute to sarcopenia phenotype [20–23]. Muscle strength generally increases with growth in young adulthood(up to 40 years), maintains during midlife, and decreases with aging [23]. Therefore younger adults must be included in future discussions on sarcopenia for early detection and intervention to prevent any critical accidents in their later years of life. Previously, a population-based study suggested that sarcopenia is a risk factor for age-related hearing loss in Korean people [24]. Meanwhile, postural dysfunction in sarcopenia was explored in only a limited number of studies on elder individuals [4, 25, 26]. The objective of this paper was to investigate the association between the prevalence of sarcopenia and postural dysfunction in a wide age range of adults using data from a nationally representative cohort study in Korea.

## Materials & methods

### Study population

**Fig 1:**
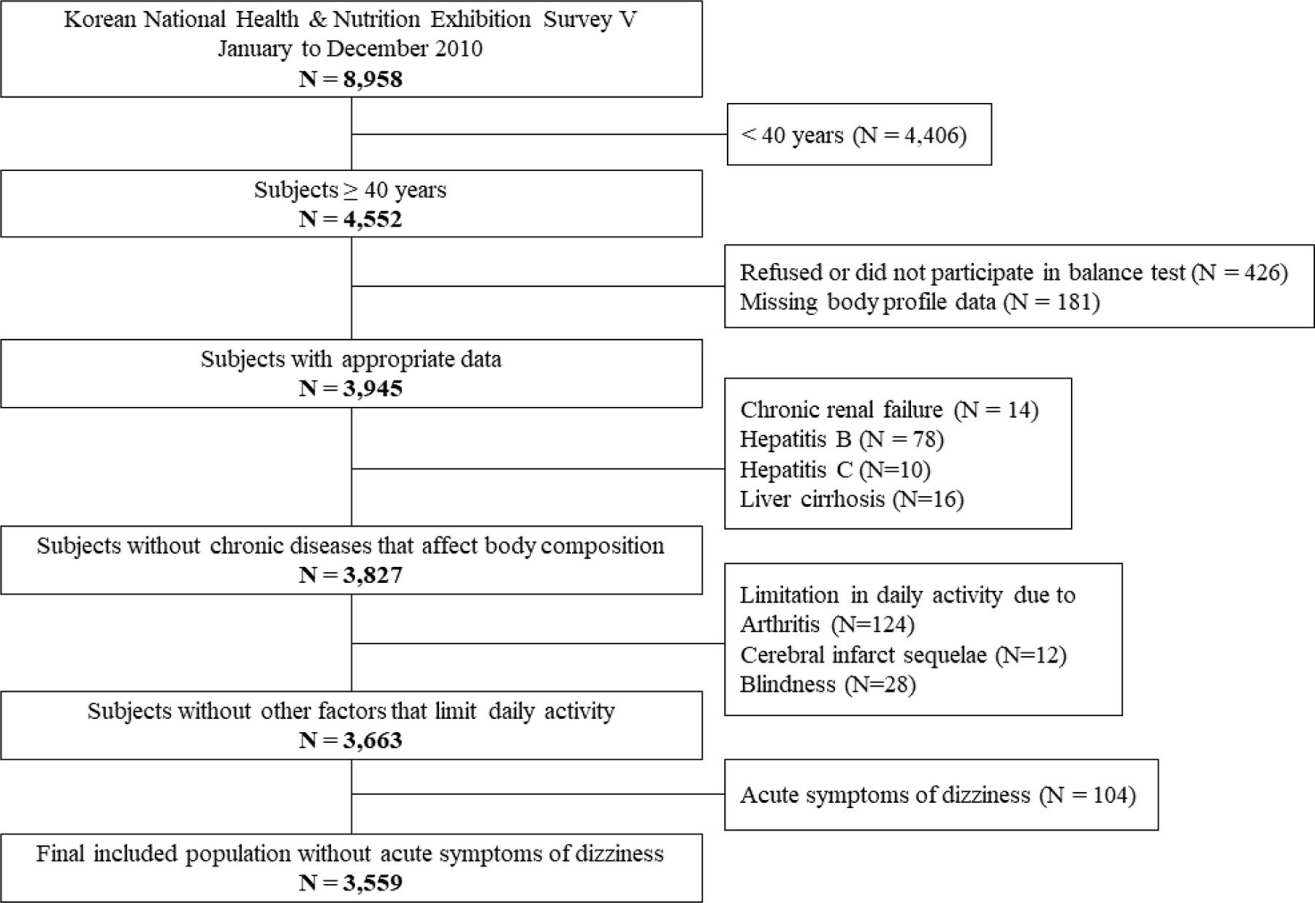
Flow diagram demonstrating participant selection process.

Korean National Health & Nutrition Exhibition Survey V (KNHANES V, 2010–2012) is the fifth cross-sectional survey of the South Korean population (51.6 million) performed by the Korean Ministry of Health and Welfare [27]. This nationwide survey includes health/nutrition interviews and health examinations. The study population is selected from the total South Korean population using a rolling sampling method. The acquired data has been kept open to the public by the Korea Center for Disease Control and Prevention (KCDC). The study described here adhered to the tenets of the Declaration of Helsinki, and written informed consent was obtained from all participants. The survey protocol was approved by the Institutional Review Board of the KCDC (IRB No: 2010-02CON-21-C). Due to the low prevalence of sarcopenia in young adults and children, subjects younger than 40 years were excluded. From a total of 8,958 subjects sampled from January to December 2010, 4,552 subjects were 40 years or older. Those who refused or did not participate in the balance test (N = 426) and those with missing body profile data (N = 181) were excluded. We also excluded subjects with chronic renal failure (N = 14), hepatitis B (N = 78), hepatitis C (N = 10), and liver cirrhosis (N = 16), since these diseases can affect body composition. Subjects that had limitation in daily activity due to arthritis (N = 124), cerebral infarct sequelae (N = 12), and blindness (N = 28) were also excluded. According to a symptom questionnaire, 104 subjects with acute symptoms of dizziness were excluded. Finally, 3,559 subjects were enrolled in the study (Fig 1).

### Assessment of sarcopenia

Total appendicular mass, appendicular fat tissue mass, and appendicular bone mass were measured in kilograms using dual-energy X-ray absorptiometry scans (DXA) (QDR 4500A, Hologic Inc., Waltham, MA, USA). Appendicular skeletal muscle mass (ASM)/height (ht)^2^ was calculated using the formula:

$$\frac{Total\;appendicular\;mass-Appendicular\;fat\;tissue\;mass-Appendicular\;bone\;mass}{{Height}^{2}}$$

Based on the value of ASM/ht^2^, participants were classified into a non-sarcopenic group or a sarcopenic group. In this study, sarcopenia was defined according to the cutoff values <7.0 kg/m2 in men and <5.4 kg/m2 in women [28].

### Assessment of postural dysfunction

The foam balance test was performed in all participants of the KNHANES to evaluate the prevalence of postural dysfunction. A 50×50×12-cm polyurethane sponge (density 22 kg/m^3^) was used and replaced every three months (approximately 1,000 tests). Selected test examiners were trained annually by the Epidemiologic Survey Committee of the Korean Otolaryngologic Society to perform the test. Audit committee members provided performance monitoring on a regular, periodic basis. They visited test examiners to observe them performing the balance examination and verify that standard testing procedures were being strictly followed. Exclusion rates and outcome statistics were continually monitored, and retraining was conducted as necessary.

**Fig 2:**
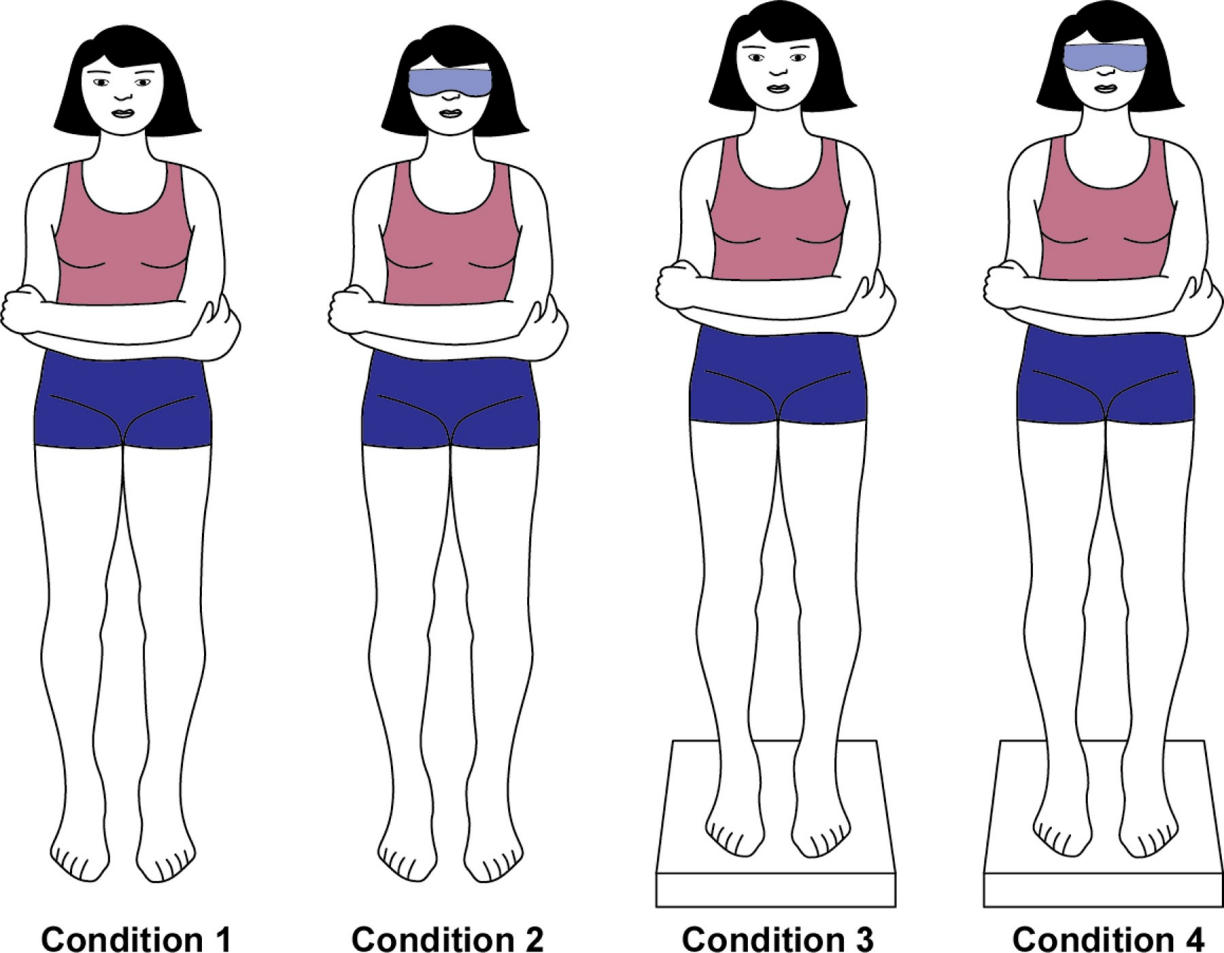
Modified Romberg test using a foam pad (“foam balance test”).

Subjects were instructed to stand with feet 10 cm apart, and arms folded across the waist, holding elbows with the hands. They were told to look at a target 1–2 m away. The duration until they lost balance (falling, side-stepping, hopping, pivoting, etc.) was measured in seconds (Fig 2). If the test subject was able to maintain their stance for 15 seconds, they “passed” the test. If the test subject was unable to maintain their stance for 15 seconds, they were offered a second chance. If the examinee refused or failed the second trial, they were classified as “did not pass.”

All participants performed the test under four different conditions. Condition 1 was tested as described above. Condition 2 was identical to Condition 1 except that the test subject was ordered to keep their eyes closed. Conditions 3 and 4 are performed as Conditions 1 and 2, respectively, but the examinee was told to stand on a FOAM pad and was tested for 20 seconds.

Condition 1 allows the subject to use all sensory inputs (vision, vestibular function, and proprioception) to maintain their balance and thus is the easiest task. Condition 2 eliminates visual input, and the subject can only rely on vestibular and proprioceptive information. In Condition 3, the subject must maintain balance on a foam surface, which limits proprioception. In Condition 4, both visual and proprioceptive input is limited; thus, the subject must rely only on their vestibular function.

All subjects performed all four trials regardless of whether they failed the previous conditions. We defined subjects who passed Condition 4 (regardless of failure in the previous conditions) as those who passed the foam balance test and regarded them as “normal.” Subjects who did not pass Condition 4 were defined as those who “failed” the foam balance test because of postural dysfunction.

Prior to the exam, an interview was held to exclude those who cannot (or do not need to) perform the test. Those who were unable to stand unassisted, have any prosthetic device below the waist, have bodyweight exceeding 120 kg, have current symptoms of severe dizziness or lightheadedness, refused the test, or were under 40 years old were excluded.

### Statistical analysis

Clinical characteristics listed in Table 1 are presented as mean±standard error (SE), and t-tests or χ^2^ tests were done, respectively. The association between sarcopenia and postural dysfunction was analyzed using Pearson’s χ^2^ test. A logistic regression model was used to further assess this relationship. The odds ratios (OR) and 95% confidence interval (CI) were calculated. Using a stepwise regression method, the OR was adjusted to filter for two factors, age, and sex. Further analysis was done in subgroups according to sex and age (< 65 years and ≥65 years). *P*-values < 0.05 were accepted as statistically significant results. All statistical analyses were performed using Statistical Package for the Social Sciences (SPSS) software (version 20.0, SPSS Inc., Chicago, IL).

**Table 1:** Clinical characteristics of the study population.

	Male		P-value	Female		P-value	Sarcopenia		P-value
	Normal	Sarcopenia		Normal	Sarcopenia		<65yrs	≥65yrs	
	N = 1,108	N = 480		N = 1,442	N = 529		N = 636	N = 373	
Age (yr)	55.8±10.8	62.2±11.9	<0.001*	57.1±10.9	56.9±12.1	0.739	51.5±7.1	73.0±5.4	<0.001*
BMI (kg/m^2^)	25.0±2.5	21.4±2.1	<0.001*	24.7±3.0	21.5±2.3	<0.001*	21.4±2.1	21.5±2.4	0.307
Waist circumference (cm)	87.1±8.0	80.0±7.5	<0.001*	82.2±8.8	75.4±7.9	<0.001*	76.3±7.7	79.8±8.1	<0.001*
Total body fat (%)	23.3±5.1	23.2±5.6	0.828	34.4±5.3	35.0±5.5	0.028*	30.1±7.8	28.2±8.5	0.001*
ASM/Ht^2^ (kg/m^2^)	7.9±0.6	6.5±0.4	<0.001*	6.1±0.6	5.0±0.3	<0.001*	5.6±0.8	5.9±0.8	<0.001*

Data are presented as mean±standard error(SE)

Abbreviations: yrs, years; BMI, body mass index; ASM, appendicular skeletal muscle mass; Ht, height

*Statistically significant

## Results

**Fig 3:**
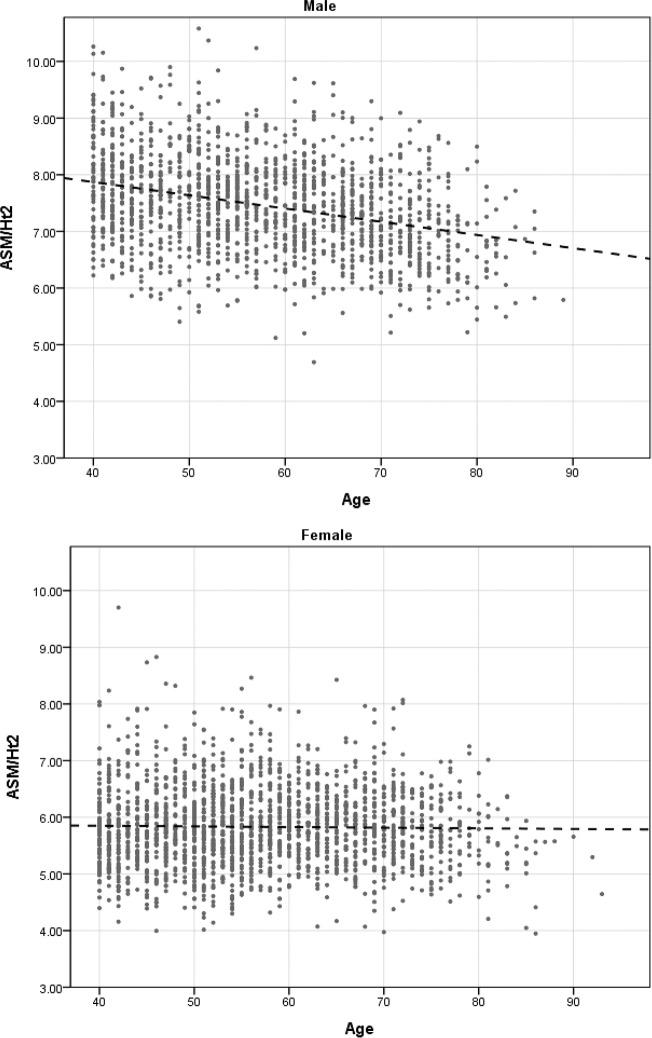
Distribution of ASM/ht^2^ according to age and sex.

The characteristics of enrolled subjects are shown in Table 1. A total of 3,559 subjects over 40 years old were enrolled (M:F = 1,588:1,971), with an average age of 57.36±11.37 years. The oldest subject was 93 years old. Among the enrolled study population, 30.2% of men and 26.8% of women were defined as having sarcopenia (95% CI: 27.9–32.5, 24.8–28.8) (*p* = 0.026). ASM/ht^2^ was significantly higher in the male population, with a calculated average of 7.46±0.85 kg/m^2^ for men and 5.83±0.70 kg/m^2^ for women (*p*<0.001). Among subjects with sarcopenia, ASM/ht^2^ was significantly higher in the old age group 65 years or older, with 5.6±0.8 kg/m^2^ for middle age and 5.9±0.8 kg/m^2^ for old age (*p*<0.001). The ASM/ht^2^ distribution in the total studied population (≥40 years old) showed that women have less muscle volume than men, but ASM/ht^2^ does not change significantly with age, while ASM/ht^2^ decreased with age in men (Fig 3).

Among the 2,550 subjects who were considered non-sarcopenic in the total studied population, 2,503 subjects (98.2%) had normal postural function, and 47 subjects (1.8%) had postural dysfunction. Among 1,009 subjects in the sarcopenic group, 963 (95.4%) passed, and 46 (4.6%) failed the foam balance test. Those with sarcopenia were significantly more likely to have postural dysfunction (*p*<0.001). Further analysis was done within each sex. In the male group, 480 subjects (30.2%) were defined to have sarcopenia, and 21 (4.4%) of them failed the foam balance test. In the female group, 529 subjects (26.8%) were defined to have sarcopenia, and 25 (4.7%) of them failed the foam balance test. A significant correlation between sarcopenia and foam balance test failure was found in both men and women (*p* = 0.001, *p* = 0.002). The studied population was divided into a middle age group (<65yrs) consisting of 2,521 subjects, and an old age group (≥65yrs) consisting of 1,038 subjects. 636 subjects (25.2%) of the middle age group and 373 subjects (35.9%) of the old age group were defined to have sarcopenia (*p*<0.001). 19 subjects (0.8%) of the middle age group and 74 subjects (7.1%) of the old age group failed the foam balance test (*p*<0.001). In the middle age group, 10 subjects (1.6%) with sarcopenia and 9 subjects (0.5%) without sarcopenia failed the foam balance test. In the old age group, 38 subjects (5.7%) with sarcopenia and 36 subjects (9.7%) without sarcopenia failed the foam balance test. In both age groups, subjects with sarcopenia were significantly more likely to have postural dysfunction (*p =* 0.006, *p* = 0.018). Among sarcopenic subjects, though the old age group had a higher ASM/ht^2^ (5.89±0.78 kg/m^2^) than the middle age group (5.63±0.81 kg/m^2^) (*p*<0.001), they were more likely to fail the foam balance test (*p*<0.001).The regression analysis revealed a significant relationship between sarcopenia and postural dysfunction with an OR of 2.544 (95% CI: 1.683–3.846 (*p*<0.001). Repeating regression analysis in the middle age group (OR: 3.330, 95% CI: 1.347–8.232) (*p* = 0.009) and the old age group (OR: 1.763, 95% CI: 1.096–2.833) (*p* = 0.019) confirmed that those with sarcopenia are more likely to fail the foam balance test. OR for sarcopenia was adjusted for age and sex, and OR for age was adjusted for sarcopenia. The adjusted ORs for sarcopenia and age were 1.746 (95% CI: 1.120–2.720) (*p =* 0.014) and 1.131 (95% CI: 1.105–1.158) (*p*<0.001). There were similar results in multivariate regression analysis in each age group, except that sarcopenia did not have sufficient significance in the old age group. In the middle age group, the adjusted ORs for sarcopenia and age were 3.344 (95% CI: 1.350–8.285) (*p* = 0.009) and (95% CI: 1.029–1.188) (*p* = 0.006). In the old age group, the adjusted ORs for sarcopenia and age were 1.302 (95% CI: 0.791–2.142) (*p* = 0.299) and 1.155 (95% CI: 1.107–1.206) (*p*<0.001) (Table 2).

**Table 2:** Adjusted Odds Ratio (aOR) and 95% Confidence Intervals (CI) for postural instability related to sarcopenia, age, and sex.

	All Subjects		<65yrs		≥65yrs	
	aOR (95% CI)	*P-value*	aOR (95% CI)	*P-value*	aOR (95% CI)	*P-value*
Male	Reference		Reference		Reference	
Female	1.158 (0.770–1.780)	0.460	1.058 (0.424–2.639)	0.904	1.350 (0.834–2.183)	0.222
Age	1.137 (1.111–1.164)	<0.001*	1.105 (1.029–1.187)	0.006*	1.160 (1.112–1.210)	<0.001*
Age (- Sarcopenia)	1.131 (1.105–1.158)	<0.001*	1.106 (1.029–1.188)	0.006*	1.155 (1.107–1.206)	<0.001*
Normal ASM/ht^2^	Reference		Reference		Reference	
Sarcopenia	2.544 (1.683–3.846)	<0.001*	3.330 (1.347–8.232)	0.009*	1.763 (1.096–2.833)	0.019*
Sarcopenia (- Age, Sex)	1.747 (1.120–2.720)	0.014*	3.344 (1.350–8.285)	0.009*	1.302 (0.791–2.142)	0.299

Abbreviations: yrs, years; ASM/ht^2^, appendicular skeletal muscle mass/height^2^; CI, confidence interval; aOR, adjusted odds ratio

*Statistically significant

## Discussion

Despite a recent scientific interest in conditions that may eventually increase the risk of falls, knowledge on possible relation to sarcopenia is limited. A previous study enrolling 260 Italian adults over 80 years old showed that sarcopenic participants were over three times more likely to fall during a two year follow-up period [4]. In a study of 1,110 Japanese elderly, the odds ratio for fall in the sarcopenia group relative to the normal group was 4.42 (95%CI 2.08–9.39) in men and 2.34 (95%CI 1.39–3.94) in women [25]. There are a few articles on the association of sarcopenia and postural dysfunction, but this study is the first based on a nationally representative cohort. In our study, we further establish a substantial burden of postural dysfunction among adults with sarcopenia, with a prevalence of 1.8% in non-sarcopenic and 4.6% in sarcopenic subjects. Subjects with sarcopenia were 1.7 times more at risk of developing difficulty in postural control, regardless of age and sex. Among middle age subjects(< 65 years), those with sarcopenia were over three times more likely to have postural dysfunction. Since balance disorder is among the most common causes of falls in older adults [29], our results support previous findings that sarcopenic patients are at risk of falls.

Thus far, several operational methods for measuring muscle mass have been suggested, including ASM, ASM/ht^2^, skeletal muscle index, skeletal muscle index adjusted for fat mass along with height, weight-adjusted muscle mass index, and ASM/body mass index. The most appropriate method with the highest predictive value for identifying subjects who are at higher risk of weakness and slowness remains uncertain [30]. ASM/ht^2^ was first suggested in the New Mexico Elder Health Survey [31]. This index demonstrated significant associations with physical disability or frailty. Many research groups have used this index for defining sarcopenia, and some have reported associations between this index and many clinical outcomes [32–34]. Furthermore, guidelines provided by the European Working Group on Sarcopenia in Older People (EWGSOP) [20, 35], International Working Group on Sarcopenia (IWGS) [36], and Asian Working Group for Sarcopenia (AWGS) [28, 37] commonly recommended the use of the ASM/ht^2^ index to determine relative muscle mass. In the current study, we used ASM/ht^2^ to determine muscle mass, and used the cut off values recommended by AWGS guideline regarding that enrolled subjects were of Asian ethnicity.

The prevalence of sarcopenia was 30.2% in males and 26.8% in females. Previous studies that used the same cut off values for ASM/ht^2^ also showed male predominance (5.1%-21.0% in men vs. 4.1%-16.3% in women) [28]. The ASM/ht^2^ distribution in our study population showed that there is a stronger tendency for muscle volume to decrease with age in men. This finding supports a large prospective study in adults aged 70–79 years, which reported a change of −0.8% in appendicular skeletal mass in men over two years and no significant change in appendicular skeletal muscle mass (ASM) in women over the same period. However, a systematic review enrolling a total of 58,404 individuals highlights that findings on body composition change according to gender are inconsistent [38]. Some studies [39, 40] reported higher relative reduction of muscle mass in men than in women. The highest prevalence of sarcopenia (50%) was found among men older than 80 years, while only 43.8% of the women of the same age group corresponded to the definitions of sarcopenia [31, 41, 42]. In contrast, other studies yielded high rates of sarcopenia among women younger than 80 years [42].

In the current study, a higher percentage of males were defined to have sarcopenia than females, and those with sarcopenia were significantly older. In the female group, there was no significant difference in age among the sarcopenic and non-sarcopenic group. Our results suggest that men may be more prone to muscle loss. Nevertheless, not much is known about potential sex differences in body composition change with aging. After the eighth decade of life, testosterone concentrations rapidly decline in human males, which may possibly explain the decrease in lean body mass and according increase in sarcopenia [38]. However, despite the suspicious gender difference in sarcopenia prevalence, each sex group revealed that sarcopenic subjects were more likely to have postural dysfunction. Linear regression results reassure that sex is not a risk factor for postural dysfunction.

The Modified Romberg test using a foam pad (“foam balance test”) is a simplified method of sensory organization test (SOT), which measures the duration between when a patient stands on a soft plastic pad and until they fall off [43–45]. In order to perform a large population-based study, the foam balance test was a suitable method that can be easily administered in all clinical settings [46]. Previous studies support that the foam balance test is a suitable method of screening adults for vestibular disorders [47, 48]. There is a report that the foam balance test correlates well with SOT condition 5 as an objective measure of balance [49]. The reliability of the foam balance test was reported with an interclass correlation coefficient (ICC) of 0.98, indicating that the foam balance test is a useful test to assess balance deficits in older adults [50]. The foam balance test is reported with excellent reliability and validity in adults with vestibular disorders [46]. However, we are unaware of any reports of reliability and validity supporting the use of foam balance test in young individuals or individuals with sarcopenia. Therefore, the generalizability of the foam balance test is limited.

Recent findings support that sarcopenia develops at younger periods of life, and sarcopenic features may begin to occur in younger adults [20, 28]. This study is the first to investigate the association between sarcopenia and postural dysfunction among both middle age and older adults, including a wider spectrum of sarcopenic populations. Our results show that sarcopenia is a more potent risk factor for balance problems in younger adults compared to the elder. We assume postural dysfunction is caused in a more multifactorial manner in elderly, possibly relating to other consequences of aging. It is well established that sarcopenia accompanies age-related decrease of muscle mass. Therefore when adjusted by age, sarcopenia does not seem as a strong risk factor in the elderly. However, in middle age subjects, whose sarcopenic changes have just begun, a decrease in muscle mass should be taken as a serious predictive factor for balance problems and potential fall accidents.

This study reveals that sarcopenia and age are independent risk factors for postural dysfunction. The decline in the strength of skeletal muscle and cross-sectional muscle area is known to affect performance in old age populations [51, 52]. Suggested mechanisms related to age are the decline of sex hormone secretion, apoptosis, and mitochondrial dysfunction [35]. Maintaining muscle volume is not only related to motor power but is also an essential factor in maintaining posture, with regards to coordination. Muscle coordination training, such as tai-chi and dancing, in combination with a sufficiently high protein diet (1.2–1.5 g/kg body weight/day), can improve balance in elderly adults and prevent fall-related accidents [53, 54]. Accordingly, we may assume that functional status reversibly improves when muscle volume is increased.

Interestingly, among those defined as sarcopenia, while subjects of older age had higher ASM/ht^2^, more of them failed the foam balance test. In accord with our results, a large scale longitudinal study by Goodpaster et al. [55] reported that during aging, muscle strength deteriorates more rapidly and to a greater extent than muscle mass, and this divergence is suggestive of an aging-related loss of muscle quality. Likewise, we can hypothesize that in addition to muscle quantity, muscle quality may be another critical determinant of loss of muscle function and balance with aging. Further studies are required to explore the deterioration of muscle strength in the elderly, and its relation with postural control, so that more targeted interventions may be planned to prevent and reverse postural dysfunction.

In our study, we provide a strong pool of evidence for the substantial burden of postural dysfunction among adults with sarcopenia, with a prevalence of 28.3% among 3,559 subjects. This prevalence is considerably higher than estimates in previous studies. A recent review of epidemiology studies from Asian countries reports the prevalence of sarcopenia ranging from 5.5% to 25.7% [28]. Guidelines provided by the EWGSOP, EWGSOP2, and AWGS commonly recommend a diagnosis algorithm which assesses not only muscle mass but also muscle strength and physical performance. Commonly grip strength and gait speed are used to evaluate these factors. Since neither were available from KNHANES data, our definition of sarcopenia was strictly based on appendicular mass calculations based on DXA scans, inevitably overestimating the prevalence of “sarcopenia.” Our results include those that are not determined by conventional diagnosis criteria but are in early stages or at risk of developing sarcopenia. Rather than identifying the true prevalence of sarcopenia, this study intends to include all data identifying sarcopenia frequency as a whole national wide population. Since this study aims to evaluate the association between decreased muscle volume and postural dysfunction, our results gain value by adopting a broader definition of sarcopenia.

A fundamental limitation of our study is that the results are not based on longitudinal studies of muscle mass and balance function over time. Many elderly persons have multiple factors that may cause dizziness and unsteady gait. Common factors are sensory deficits, such as bilateral vestibular failure, polyneuropathy, and impaired visual acuity; benign paroxysmal positioning vertigo; and central disorders, such as cerebellar ataxia and normal-pressure hydrocephalus [8]. Our study excluded those with acute symptoms of severe dizziness or lightheadedness, those with limitation in daily activity due to arthritis, cerebral infarct sequelae, and blindness. Though other factors such as lower extremity peripheral neuropathy still may have caused an overestimation of the prevalence of postural dysfunction, since KNHANES V excludes participants with gait abnormality, we assume these factors were excluded already. However, our results were generally consistent with previous studies [11, 12, 16–18] but with a larger sample size, demonstrating a strong association between sarcopenia and postural dysfunction after adjusting for multiple factors. This study analyzed the relationship between the prevalence of postural dysfunction and sarcopenia among the Korean general population using national data. Sarcopenia can be regarded as a precipitating factor for postural dysfunction. Our results may explain the increase of falling in old adults and predicting those with a high risk of postural dysfunction. Managing such high-risk individuals with caution can ultimately prevent severe injury or death caused by falling accidents.

## Conclusions

Our study reveals that sarcopenia nearly doubles, and more than triples in middle age adults younger than 65 years old, the risk of postural dysfunction. Sarcopenia significantly correlates with the prevalence of postural dysfunction, and this relationship is independent of the known risk factors for postural instability, age, and sex. Postural dysfunction is a critical risk factor of fall accidents in the elderly. Paying attention to and making efforts to prevent the sarcopenic condition, even in younger adults, can help improve the quality of daily life and prevent severe injuries.
